# Succession in arbuscular mycorrhizal fungi can be attributed to a chronosequence of *Cunninghamia lanceolata*

**DOI:** 10.1038/s41598-019-54452-z

**Published:** 2019-12-02

**Authors:** Nini Lu, Xuelei Xu, Ping Wang, Peng Zhang, Baoming Ji, Xinjie Wang

**Affiliations:** 1 College of Forestry, Beijing Forestry University, Beijing, 100083 China; 2 Key Laboratory for Silviculture and Conservation Joint-constructed by Province and Ministry of Education, Beijing Forestry University, Beijing, 100083 China; 3 Experimental Forest Farm, Beijing Forestry University, Beijing, 100095 China

**Keywords:** Forestry, Microbial ecology, Forest ecology, Fungal ecology

## Abstract

Arbuscular mycorrhizal (AM) fungi play an important role in plant-fungi communities. It remains a central question of how the AM fungal community changes as plants grow. To establish an understanding of AM fungal community dynamics associated with Chinese fir, Chinese fir with five different growth stages were studied and 60 root samples were collected at the Jiangle National Forestry Farm, Fujian Province. A total of 76 AM fungal operational taxonomic units (OTUs) were identified by high-throughput sequencing on an Illumina Miseq platform. The genera covered by OTUs were *Glomus*, *Archaeospora*, *Acaulospora*, *Gigaspora* and *Diversispora. Glomus* dominated the community in the whole stage. The number and composition of OTUs varied along with the host plant growth. The number of OTUs showed an inverted V-shaped change with the host plant age, and the maximum occurred in 23-year. Overall, the basic species diversity and richness in this study were stable. Non-metric multi-dimensional scaling (NMDS) analysis based on bray-curtis distance revealed that there were remarkable differentiations between the 9-year and other stages. Besides, AM fungal community in 32-year had a significant difference with that of 23-year, while no significant difference with that of 45-year, suggesting that 32-year may be a steady stage for AM fungi associated with Chinese fir. The cutting age in 32-year may be the most favorable for microbial community. The pH, total N, total P, total K, available N, available P, available K, organic matter and Mg varied as the Chinese fir grows. According to Mantel test and redundancy analysis, available N, available P, K and Mg could exert significant influence on AM fungal communities, and these variables explained 31% of variance in the composition of AM fungal communities.

## Introduction

Arbuscular mycorrhizal (AM) fungi, belonging to the phylum *Glomeromycotina*^1^, can form mutualistic associations with more than 80% of land plant species^2^. Through AM associations, plants provide carbon for the fungi and AM fungi benefit the plants with improved access to soil nutrients^3,4^, thus promoting plants’ growth and biomass production^5^. Furthermore, the fungi can improve soil physical structures and plants’ tolerance to abiotic stress, impact on nutrient dynamics and carbon cycling, and contribute to the development of more sustainable ecosystems^6–8^. Given that AM associations play a crucial role in exchanging matters between the aboveground and underground biotic communities^9,10^, understanding AM fungal community dynamics along the host plants’ growth status is essential for ecosystem management^11^.

There has been overwhelming researches that focus on how plant communities may structure AM fungal communities, and samples were required to be collected in a time series^12^. Most of the studies kept an eye on short-lived hosts. For instance, AM fungal status and communities changed as the plants communities^13^. Besides the plant communities, environmental factors including spatial distance and spatial variation may structure AM fungal communities. However, not all existing evidences support the successional changes of AM fungal communities. A study taken place in Calestienne region indicated that AM fungal communities do not follow changes in the plant community^14^, and the strength of the correlation between plant and AM fungal communities do not change as succession progressed^15^. There has also been some researches on long-lived plants influencing the AM fungal community. Liu *et al*. found that AM fungal communities did not vary across a 35-year chronosequence of *Caragana korshinskii* plantations^16^. While others thought the early successional species accelerated AM fungal colonization four times than the late successional species^17^. Researches on the relationship between woody plant and AM fungi also involves *Paraserianthes falcataria*^18^, breadfruit^19^ and willow^20^. However, compared with grassland and crops, investigations of the impact of long-lived woody hosts on the AM fungal community structure are still limited, especially collecting woody plant roots as samples.

Chinese fir (*Cunninghamia lanceolata* (Lamb.) Hook) is one of the most important timber plants in Southern China^21^. It is widely used for furniture making, bridge, boat and house building, general carpentry and timber constructions. Chinese fir has been widely planted in subtropical China to meet increasing timber demands. The area was about 8.54 × 10^6^ hectares, accounting for 21.35% of Chinese plantations. According to the economic and ecological benefits, the rotation age of Chinese fir was usually 25~30 years. However, in order to meet the rising demand for wood products, the rotation period of the Chinese fir plantations was shortened from 25–30 years to 20–25 years^22^. In some places, the plantations were harvested as young as 17 years^23^. Whether such a short rotation period is conducive to the restoration of forest soil remains to be verified. Previous studies have mainly focused on the management of the aboveground plants in the forest, the influence of soil nutrients and allelopathy on the growth of Chinese fir^24–26^. There have been limited researches on cutting age of Chinese fir, and the AM fungi community dynamic was lack.

The main aim of our study was to understand the dynamic of the AM fungal community associated in a chronosequence of Chinese fir, explore the driving environmental factors and provide a foundation for cutting age of Chinese fir. To achieve that, 60 samples were obtained from Chinese fir stands with five different growth stages and some questions were raised: Do the AM fungal communities change along a chronosequence of Chinese fir? If so, which stage (period) does the variation occurs? Which environmental factors are most closely related to the variation? How long is the best rotation period?

## Materials and Methods

### Study area and sampling

The study was conducted at the Jiangle state-owned Forest Farm (E117°05′~117°40′, N26°26′~27°04′), Fujian Province, China. This area is low hilly land with an average elevation of 258 m. The site is located in a typical subtropical climatic zone, with an annual mean temperature of 19.8 °C and an annual mean precipitation of 1684 mm. The dominant trees are *Cunninghamia lanceolata* Lamb, *Pinus massoniana* L. and *Phyllostachys pubescens* with a forest coverage rate of 84.5%. The soil is red soil according to Chinese soil classification^27^.

**Figure 1:**
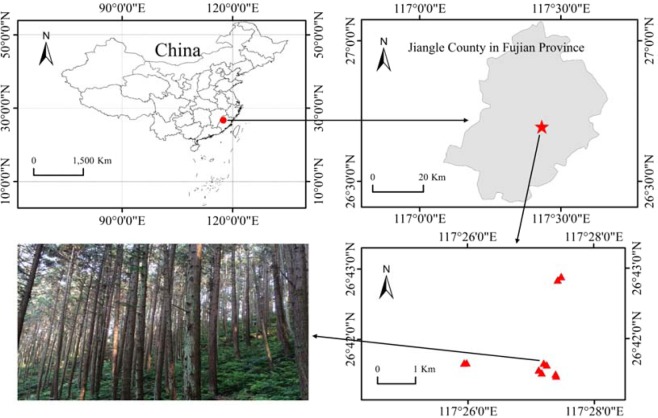
Map of the sampling sites. The red dots were the location of sample sites. The photograph of the trees was taken by the author, Nini Lu.

According to the distribution of Chinese fir in the forest farm, Chinese fir with five growth stages: 9-year, 17-year, 23-year, 32-year, 45-year were selected (Fig. 1). Two standard sites with 20 × 30 m^2^ were set up for each age group (the basic information of each standard site was shown in Table 1). Six trees were selected as the research objects in each survey standard site, and 60 trees were got. The sources of all the sample trees were: Chinese fir seeds were collected from local seed orchards, and planted in local nurseries. After growing for one year, the seedlings were transplanted to the mountains for afforestation. The soils in nurseries were local red loam, and the soils were disinfected by potassium permanganate before sowing Chinese fir seeds. Sampling was conducted in April 2015. Fine roots and rhizosphere soil (20 cm in diameter and 20 cm in depth) were collected in each sample tree from four directions (east, south, west and north), and then mixed for subsequent analysis. Finally, 60 root samples and 60 soil samples were obtained. The soil samples were sieved with a 2-mm sieve, transported to the laboratory for further processing.

**Table 1:** The basic information of each standard site.

Age of stands	altitude/m	slope/°	aspect
9-year	181	30	semi-sunny
9-year	181	31	semi-sunny
17-year	251	28	sunny
17-year	243	30	semi-shady
23-year	211	23	semi-sunny
23-year	230	27	semi-shady
32-year	254	34	semi-shady
32-year	235	32	semi-shady
45-year	200	29	semi-sunny
45-year	200	29	semi-sunny

### Root Processing, Deoxyribonucleic Acid (DNA) Extraction and Polymerase Chain Reaction (PCR) Condition

Fresh fine roots (length = 1 cm) of individual tree were cleaned with distilled water and prepared for DNA extraction using a modified protocol^28,29^. Fine roots were ground with sterilized quartz sand and an extra 650 uL cetyltrimethyl ammonium bromide(CTAB) (2% (w/v), 100 mM Tris-HCl, 1.4 M NaCl, 20 mM EDTA, pH = 8.0) was added and incubated in a 65 °C water bath for 30 min with occasional shaking. 650 uL chloroform/isoamyl-alcohol solution (24:1) was added to each tube and shaken thoroughly to form an emulsion. The mixture was spun at 12,000 g for 15 min at 25 °C in a micro centrifuge and the supernatant phase decanted into a fresh 1.5 mL tube. Supernatant containing DNA was re-extracted with chloroform/isoamyl-alcohol solution (24:1) at 4 °C until no interface was visible. Thirty uL 5 M KAC was added into the supernatant followed by 200 uL isopropanol and inverted gently to mix. The genomic DNA was precipitated at 9200 g for 2 min at 4 °C in a micro centrifuge. The DNA pellet was washed with 70% ethanol twice and dried using SpeedVac ^®^ (AES 1010; Savant, Holbrook, NY, USA). The DNA pellet was then re-suspended in 65 uL sterile deionized water and stored at -20 °C.

Genomic DNA was amplified with nested polymerase chain reaction (PCR) for Illumina Miseq sequencing. GeoA2^30^/AML2^31^ were used as the primers in the first amplification, while AMDGR^32,33^/NS31^34^ were chosen in the second amplification, and 12 np unique barcode was added at the 5′-end of NS31. The first PCR mixture (25 uL) contained 12.5 uL 2 × Taq PCR MasterMix (Tiangen Biotech Co. Ltd, Beijing, China), each primer with 1 uL at 10 uM, 8.5 uL ddH_2_O, and 2 uL dilute DNA by a factor of 20, and then followed the thermal cycling: an initial denaturation at 94 °C for 3 min, 30 cycles of denaturation at 94 °C for 30 s, annealing at 48 °C for 1 min, extension at 72 °C for 3 min, and a final extension at 72 °C for 10 min. The product of the first amplification was diluted with sterilized deionized water by a factor of 100, and 2 uL of the resulting solution was used as a template for the nested PCR (50 uL). The second amplification was performed as follows: 94 °C for 3 min; 30 cycles at 94 °C for 45 s, 45 °C for 45 s, 72 °C for 1 min and 72 °C for 10 min. The PCR products were separated through a 1% agarose gel in 1X TAE. The correct bands were excised and purified with AxyPrep DNA Gel Extraction Kit (Axygen, Union City, CA, USA) and then quantified with the Nanodrop 8000 (Thermo Scientific, Wilmington, DE, USA). All purified genomic DNA were mixed with an equal molar concentration and then subjected to the Illumina Miseq platform for sequencing at the Environmental Genome Platform of the Chengdu Institute of Biology.

### Bioinformatics analyses

The raw 18S rDNA paired-end reads were assembled by FLASH-1.2.8^35^. All reads were assembled to each sample based on the specific barcodes. Sequences without ambiguous nucleotides, longer than 300 bp and average quality score >30 were retained for further analysis. The chimeras were checked and removed by “chimeras_check.py” command with usearch8^36^. The non-chimeras sequences were clustered into operational taxonomic units (OTUs) with the UPARSE at a 97% identity threshold^37^. The representative sequences were picked and assigned by blasting against the SILVA database^38^, and all non-“Glomeromycotina” sequences, as well as fewer than five reads per OTU were removed to reduce the risk of artificially inflating richness due to sequencing error^39^. The Glomeromycotina sequences were resampled to eliminate the effects of different read numbers. To further confirm the remaining OTUs belonging to AM fungi, a Neighbor joining tree was conducted in MEGA6 with the reference sequences from GenBank and Maarj*AM* using the Kimura 2-parameter model with 1000 replicates^40^. The references were chosen only if they met the followed criteria: the BLAST score >250, the query coverage >97%, the similarity between OTU and reference >97%. All representative sequences of each OTU were submitted to GenBank (accession numbers: MK685901-MK685976).

### Physical and chemical soil properties analyses

Soil pH was measured with a soil: water ratio of 1:2.5(w/v). Total nitrogen (N) and phosphorus (P) were extracted with H_2_SO_4_ + HClO_4_ and measured using a continuous flow analyzer (AA3, SEAL, Germany). Total potassium (K) and Mg were extracted with HNO_3_ + HClO_4_ and measured with atomic absorption spectroscopy (AAS, TAS-900AFG, China). Alkaline hydrolysis diffusion was used to determining alkaline-hydrolyzable nitrogen (N). Soil available phosphorus (P) was measured by the Mo-Sb anti-spectrophotometry method after being extracted with HCl-NH_4_F^41^, while soil available potassium (K) was extracted with 1 M ammonium acetate^42^. Soil organic matter was measured by the wet-oxidation method^43^.

### Statistical analyses

Shannon-wiener index, Simpson index, Chao1, Observed_otus and PD_whole_tree were calculated to compare AM fungal community’s diversity and richness among different stages, and then One-way ANOVA were performed for significant test with SPSS 18.0. Soil properties were also tested with SPSS 18.0. AM fungal community analyses were performed based on the abundance of OTUs in each sample. The AM fungal community composition was subjected to non-metric multidimensional scaling (NMDS) with the Bray-Curtis dissimilarity measurement in the package Vegan in R, and then the “anosim” function in the vegan package was carried out to test whether there were significant AM fungal community composition differences between the different stages. Redundancy analysis (RDA) was performed to obtain the explanatory soil variables most related to the AM fungal community with vegan package in R^44^. Then the mantel and partial mantel test were used to test whether there were significant relationships between the AM fungal communities and environmental factors^45^.

## Results

### 18S rDNA sequencing analysis

**Figure 2:**
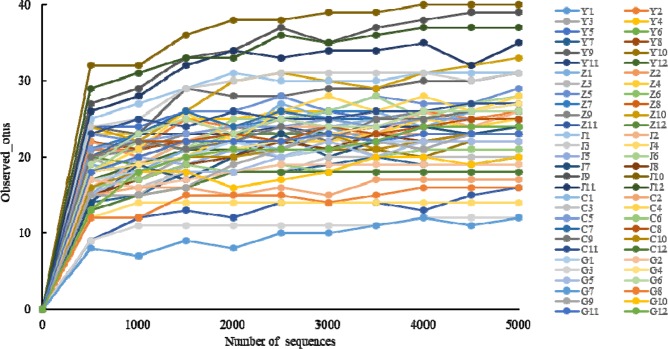
Rarefaction curves of Observed_otus numbers of all the samples from five growth stages after rarefied. When the rarefaction reached 3000, Observed_otus numbers tended to be stable. This indicated that rarefaction concentration of sequences do have a strong impact on the results, while the strength of influence become weaker when the rarefaction reaches a certain degree. The Y1-Y12 represented samples in 9-year, Z1-Z12 represented samples in 17-year, J1-J12 represented samples in 23-year, C1-C12 represented samples in 32-year, G1-G12 represented samples in 45-year.

A total of 1111 828 sequences were retained from raw reads. A total of 1098 554 reads were clustered into 199 OTUs with UPARSE. Among these sequences, 1060 007 sequences (96.5% of total) which means 77 OTUs belonged to *Glomeromycotina* (after being assigned with the SILVA database). After removing sequences fewer than five from the data set, 1059 992 sequences were remained. Rarefaction curves were constructed showing the number of observed OTUs (Fig. 2). When the number of sequences increased from 0 to 500, the number of observed OTUs increased sharply. Since then, as the number of sequences increased, the rate of increase in the number of observed OTUs slowed down. And when the number of sequences reached 5000, the obserbed OTUs were nearly saturated. Therefore, 5000 sequences were retained for each sample in the resampleing step. A total of 300 000 sequences were resampled to eliminate the effects of different sequences numbers. By comparing with the references sequences from GeneBank and *MaarjAM* through the Neighbor Joining tree, 76 OTUs were retained for further analysis (see the Supplementary Figure A1).

The genera covered by OTUs were *Glomus*, *Archaeospora*, *Acaulospora*, *Gigaspora* and *Diversispora*. Among the 76 AM fungal OTUs, 57 OTUs belonged to *Glomus*, 11 OTUs belonged to *Archaeospora*, 5 OTUs belonged to *Acaulospora*, 1 OTUs belonged to *Gigaspora*, and two OTUs belonged to *Diversispora*. From 9-year to 17-year, the number of *Glomus* OTUs increased from 31 to 51 (an increase of 64.5%), and then began to decline. When it reached 45-year, the number of *Glomus* OTUs was 39. The number of *Archaeospora* OTUs decreased in the whole stages. The maximum appeared in the 9-year (that was 10), and the minimum appeared in the 32-year (that was 4). From 23-year to 45-year, there was no significant difference in the number of *Archaeospora* OTUs (which was 5-4-5). The trend of *Acaulospora* OTUs was “V”-shaped. The number was biggest in the 9-year (that is 4), and smallest in the 17-year and 23-year (both were 2). Compared with other genera, the number of *Gigaspora* OTUs was always 1. *Diversispora* only appeared in the 23-year, and the number of *Diversispora* OTUs was 2.

**Figure 3:**
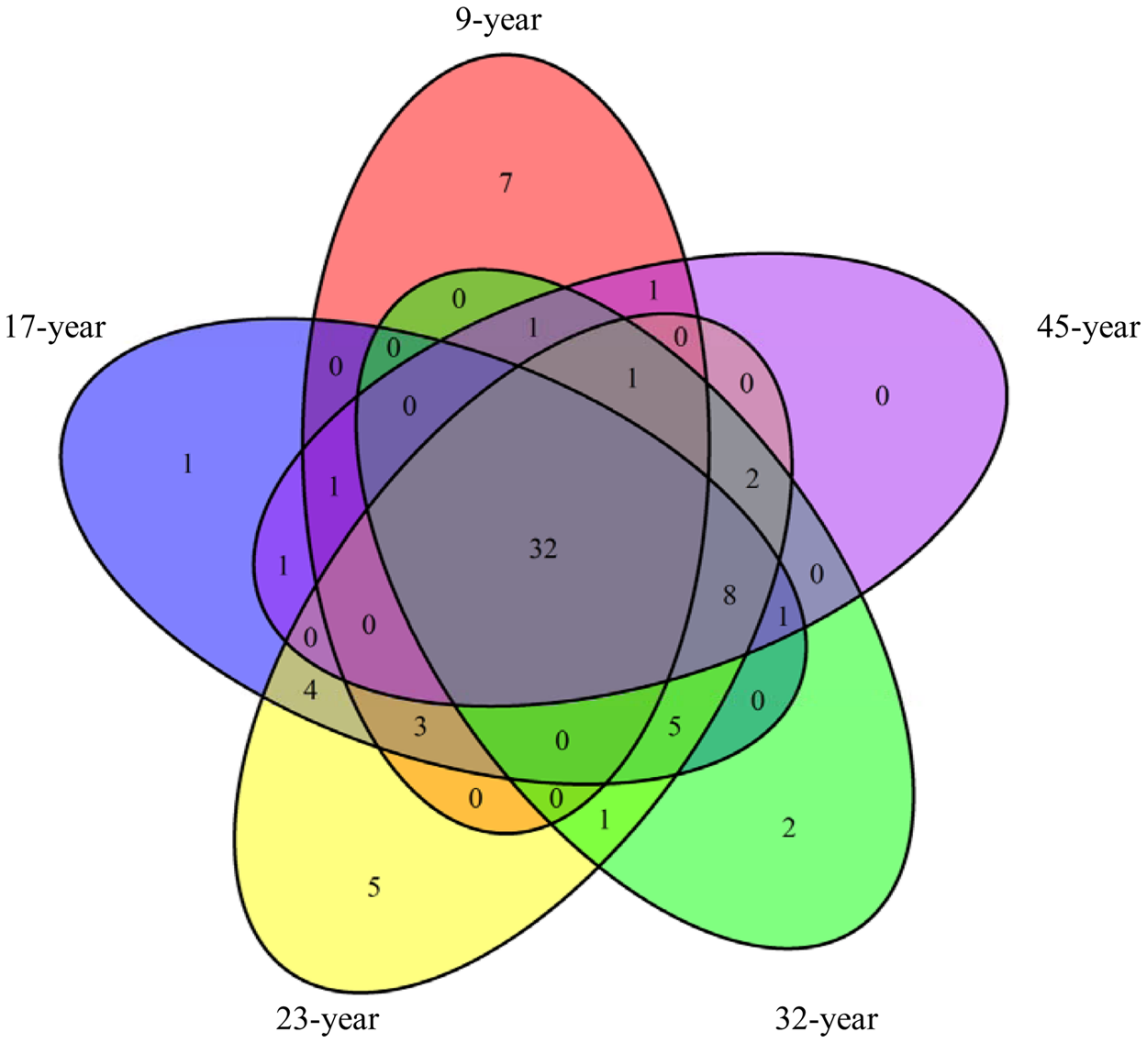
Venn diagrams showing the number of OTUs that were unique to and shared between different growth stages.

There was a maximum (61) of OTUs in the 23-year. 46 OTUs were from the trees in the 9-year, 56 OTUs from the trees in 17-year, 61 OTUs from the trees in 23-year, 53 OTUs from the trees in 32-year, and 48 OTUs from the trees in 45-year. 32 OTUs were occupied by the trees in all the growth stages, while 15 OTUs were unique (Fig. 3).

### AM fungal community composition

**Figure 4:**
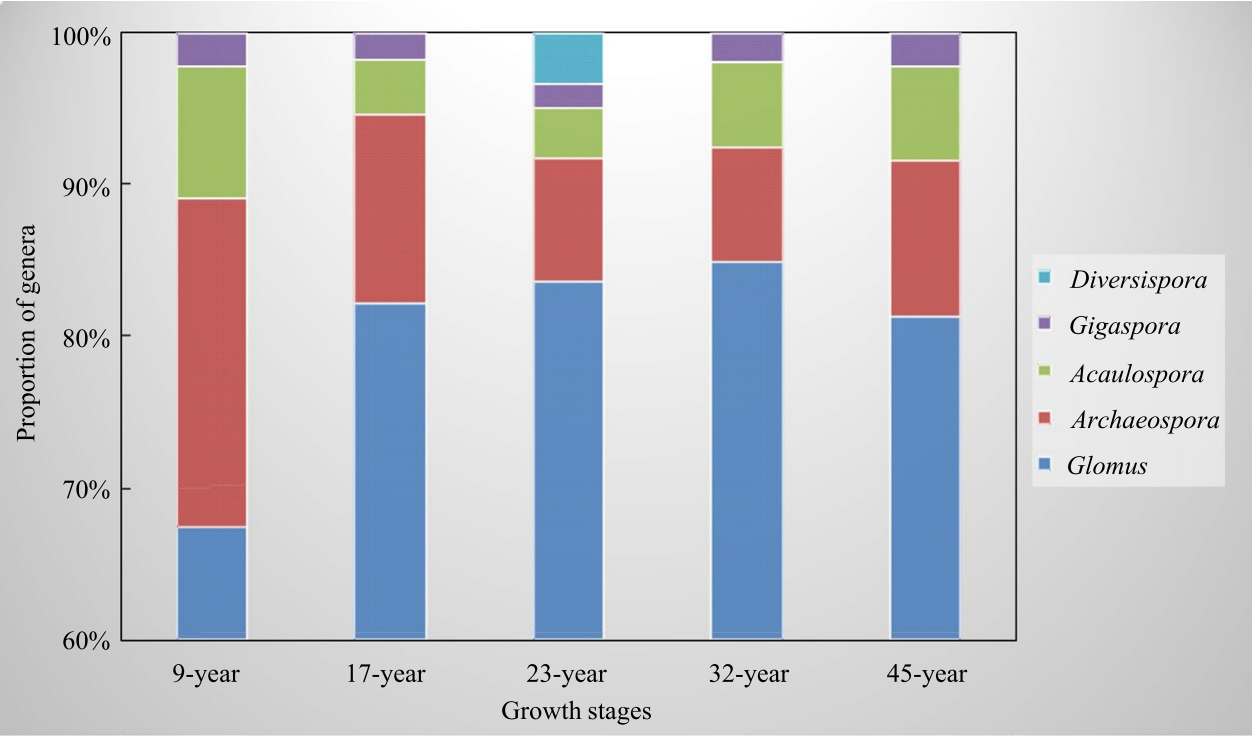
Proportion of five arbuscular mycorrhizal (AM) fungal genera in different stages.

There were four orders, five families and five genera in the samples. *Glomus* dominated the community in the whole stage, the second was *Archaeospora*. While *Diversispora* only existed in the 23-year stage. The proportion of *Glomus* increased from 67% to 85% when the host trees growed from 9-year to 32-year stage, while the *Archaeospora* proportion decreased from 22% to 7% as the host plant grows. The *Acaulospora* proportion decreased from 9-year to 23-year, and turned to increase after 23-year. The minimum proportion of *Acaulospora* was appeared in the 23-year with 3%. The proportion of *Gigaspora* was always 2% in the whole stages (Fig. 4).

**Figure 5:**
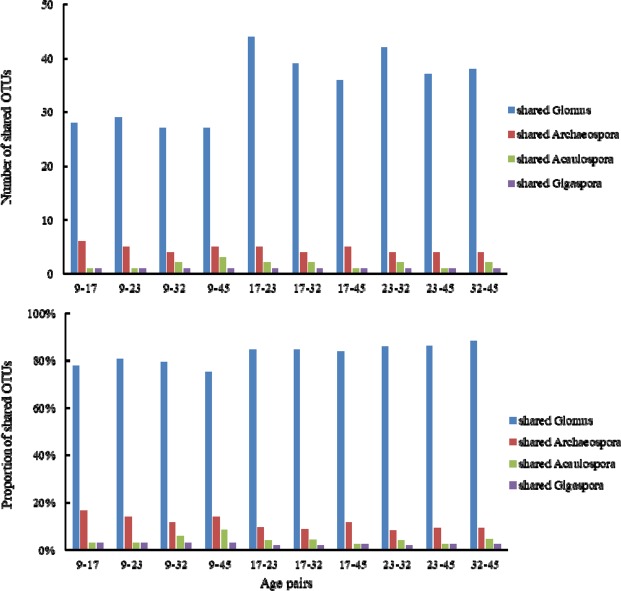
The number and proportion of shared OTUs in each age pair.

When we compared the composition of shared OTUs, the number of shared OTUs had a minimum in 9–32 year (that’s 34), and the maximum was 17–23 year (that’s 52) (Fig. 5). Among these common OTUs, *Glomus* accounted for the largest proportion (≥75%), followed by *Archaeospora*, *Acaulospora* and *Gigaspora*. The proportion of shared OTUs in each pair varied from 51% to 80%. The number of shared OTUs between 9-year and other growth stages were less than 40, suggesting that the composition in 9-year had significant difference with other growth stages. The number of shared OTUs between 17–23 year were over 50, suggesting that the difference was the smallest. The number of shared OTUs between 32-year and 9-year, 17-year, 23-year steadily increased (from 34 to 46 and 49), while decreased in 45-year, indicating the composition difference increased from 9-year to 23-year, then decreased. The variety of the *Glomus* composition was similar to that of shared OTUs, indicating that the variety of *Glomus* decided the whole variety of AMfungi. When it terms to the proportion of composition of each shared genus, the biggest difference appeared in the 9–17 years, and the smallest difference appeared in the 9–45 years.

**Figure 6:**
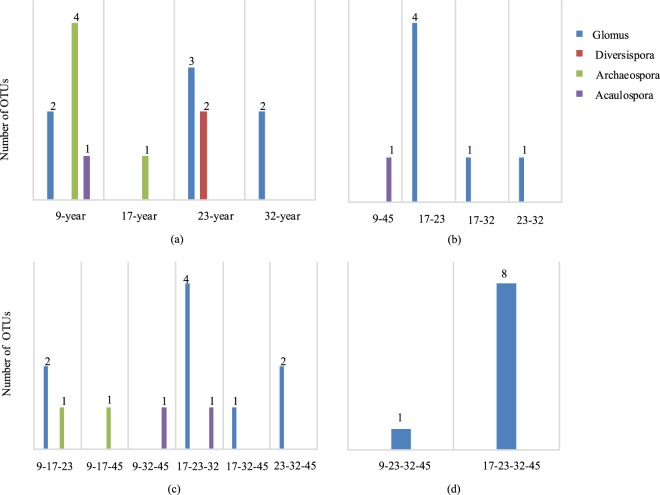
The number of unique OTUs in each growth stage. (**a**) showed the number of OTUs only appeared in one growth stage, 9-year means the OTUs appeared in 9-year, and so on. (**b**) showed the number of OTUs appeared in two growth pair, 9–45 means the OTUs appeared in both 9-year and 45-year, and so on. (**c**) showed the number of OTUs appeared in three growth pair, 9-17-23 means the OTUs appeared in 9-year, 17-year and 23-year, and so on. (**d**) showed the number of OTUs appeared in four growth pair, 9-23-32-45 means the OTUs appeared in 9-year, 23-year, 32-year and 45-year, and so on.

With regard to unique OTUs, the number of OTUs only appeared in one growth stage was 15 (Fig. 6a), among those 2 OTUs belonged to *Diversispora*, 5 OTUs belonged to *Archaeospora*, 1 OTU belonged to *Acaulospora*, 7 OTUs belonged to *Glomus*. The number of OTUs appeared in two growth stages was 7 (Fig. 6b), among those 6 OTUs belonged to *Glomus*, 1 OTU belonged to *Acaulospora*. The number of OTUs appeared in three growth stages was 13 (Fig. 6c), among those 2 OTUs belonged to *Acaulospora*, 2 OTUs belonged to *Archaeospora*, 9 OTUs belonged to *Glomus*. The number of OTUs appeared in four growth stages was 9 (Fig. 6d), all of those OTUs belonged to *Glomus*.

**Figure 7:**
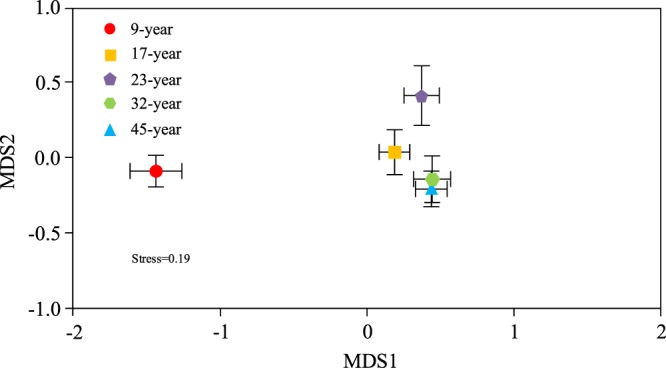
Non-metric multidimensional scaling (NMDS) of arbuscular mycorrhizal (AM) fungal communities associated with a chronosequence of *Cunninghamia lanceolata* (Chinese fir). The difference between communities based on Bray-Curtis. Each point represents the centroid of the AM fungal community of each stage with vertical and horizontal bars depicting ± SE.

Non-metric multi-dimensional scaling (NMDS) analysis based on bray-curtis distance showed the differences in the AM fungal community composition between the different stages (Fig. 7). The pattern was verified by the anosim results (Table A1). There were remarkable differentiations between the 9-year and other stages (*p* < 0.01). There were also significant differences between the 23-year/32-year, and 23-year/45-year (*p* < 0.05). But there were no significant differences between 32-year and 45-year.

AM fungal community’s diversity and richness changes as the host plant grows (Table 2). The Shannon-wiener and Simpson indexes indicated that the most diverse AM fungal community was in the 9-year. Chao1 and Observed_otus showed that the number of OTUs was largest in the 23-year. The PD_whole_tree was greatest in the 23-year. There were significant differences between 9-year and 45-year in the matter of Shannon and Simpson indices. There also has significant difference between 23-year and 45-year of Observed_otus. The index of PD_whole_tree was maximum is 23-year indicating that the AM fungal community composition was the most complex in 23-year, this is consistent with that there were the most genera in 23-year.

**Table 2:** Estimated richness and diversity indices (mean ± standard error) for the fungal communities associated with Chinese fir in different stages.

growth stage	Shannon	Simpson	Chao1	Observed_otus	PD_whole_tree
9-year	3.06 ± 0.10a	0.81 ± 0.02a	25.12 ± 0.79a	24.50 ± 0.81a	1.12 ± 0.03a
17-year	2.80 ± 0.19ac	0.75 ± 0.05ac	29.40 ± 2.23a	25.25 ± 1.16a	1.07 ± 0.05a
23-year	2.94 ± 0.15ac	0.79 ± 0.02ac	32.30 ± 2.71a	29.25 ± 2.24ab	1.19 ± 0.10a
32-year	2.61 ± 0.17ac	0.72 ± 0.04bc	24.65 ± 1.34a	23.42 ± 1.04a	0.92 ± 0.05b
45-year	2.38 ± 0.13bc	0.69 ± 0.03bc	23.29 ± 2.30a	20.50 ± 1.43ac	0.86 ± 0.07b

### Response of AM fungal community to soil physicochemical properties

pH, soil total nitrogen (N), total phosphorus (P), total potassium (K), available N, available P, available K, soil organic matter (SOM) and Mg were all significantly different among the stages (Table 3). There were significant difference in pH among the 9-year, 17-year and 45-year, but no significant differences between the 23-year and 32-year. It is worth mentioning that there were significant differences in total N and total P among different stages. As far as total K, available N and available P are concerned, the differences among the pairs were significant except for the difference between 23-year and 32-year. The content of available K in 9-year was significantly different from that in other stages, and the difference disappeared after 17-year. Soil organic matter (SOM) between the following pairs were significant: 9-year/17-year, 9-year/45-year, 17-year/23-year, 17-year/45-year. Mg was significantly different in the first four stages, and the difference disappeared when it reached 32-year. Generally, there were significant differences in soil physical and chemical properties between 9-year to 17-year and 17-year to 23-year.

**Table 3:** Statistical results of physical and chemical characteristics (mean ± SE) of rhizosphere soils collected from host plant with different growth stages.

Ages of stands	pH	Total N mg/kg	Total P mg/kg	Total K mg/kg	Available N mg/kg	Available P mg/kg	Available K mg/kg	SOM mg/g	Mg mg/kg
9-year	4.51 ± 0.01a	245.92 ± 3.94a	304.25 ± 3.28a	60.8 ± 0.87a	164.67 ± 6.17a	4.3 ± 0.14a	58.89 ± 0.45a	38.37 ± 0.44a	81.88 ± 0.74a
17-year	4.61 ± 0.01b	231.92 ± 3.91b	255 ± 1.30b	53.63 ± 0.76b	109.83 ± 1.80b	6.87 ± 0.26b	32.31 ± 0.57b	32.24 ± 0.44b	52.25 ± 0.37b
23-year	4.54 ± 0.06ab	214.75 ± 1.89c	281 ± 2.57c	56.08 ± 0.46c	140.5 ± 3.11c	12.15 ± 0.75c	32.26 ± 0.56b	27.63 ± 0.48a	48.29 ± 0.32c
32-year	4.54 ± 0.04ab	203.83 ± 2.11d	319.67 ± 2.73d	55.84 ± 0.57c	140.25 ± 2.23c	12.86 ± 0.61c	32.3 ± 0.44b	35.16 ± 2.14ab	46.18 ± 0.33d
45-year	4.74 ± 0.01c	179.92 ± 5.96e	389 ± 3.58e	42.41 ± 1.01d	93.69 ± 0.84d	6.04 ± 0.10d	33.19 ± 0.53b	19.20 ± 0.49c	45.17 ± 0.73d

**Figure 8:**
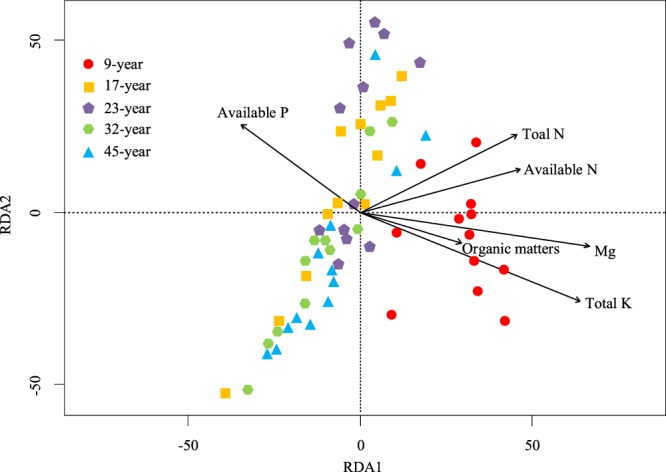
Redundancy Analysis (RDA) plot of AM fungal community composition and environmental variables among different stages.

Total N, total K, available N, available P, SOM and Mg were found to have significant influence on AMF communities based on the Mantel test (Table 4), and redundancy analysis (RDA) showed that these variables explained a total of 31% of the variance in the composition of AMF communities among stages (*F* = 2.20, *P* = 0.001, Fig. 8). During the five stages, the AM fungal community composition in 9-year (red circle) was the most relevant to soil factors. When controlling other environmental factors, total K, available N, available P and Mg were still significantly related with AM fungal community composition (*P*  < 0.05).

**Table 4:** Mantel test and partial Mantel test revealed the correlations between AM fungal community composition and soil properties.

	Mantel		Partial mantel	
	*r*	*P*	*r*	*P*
pH	0.03	0.296		
Total N	0.16	0.014	0.12	0.055
Total P	−0.08	0.936		
Total K	0.61	0.001	0.60	0.001
Mg	0.60	0.001	0.60	0.001
Available N	0.20	0.001	0.17	0.005
Available P	0.11	0.033	0.11	0.046
Available K	−0.03	0.466		
Organic matter	0.13	0.043	0.07	0.167

## Discussion

### AM fungal community changed as host plant grows

Breadfruit has been reported to have more AMF taxa in older trees than younger trees^46^. AMF species richness declined with *Populus-Salix* successional stages^47^. This difference of fungal community response may be due to different host plants. In our study, the number of AM fungal genera is most in the 23-year stage, and plant trees in 17-year had more OTUs than that of 9-year. This is in accordance with the previous research in Hunan reported that 15–20 years old Chinese fir plants had a higher infection than 5–10–year–old plants^48^.

It is interesting that AM fungal community in 9-year was significantly different from the others, while the variation disappeared when it reached 32-year. This phenomenon could be due to adequate water and nutrient for tree growth in the young period. However, as the plant trees grew faster with much fiercer competition than ever^49^, thus leading to the increased demand for effective absorption, and requiring more AM fungi to participate in absorbing more nutrients and water. When it reached the 32-year, competition tended to be steady^50^. Our results were consistent with Gordon that plants from different stages could tradeoff with nutrient gathering strategies through varying investment in AM fungi^51^. Moreover, it has been demonstrated that mycorrhizal community could evolve to suit the plants’ current properties and local ecological conditions^52,53^. Although the biomass of fine root decreased, the speed of plant growth accelerated, and the AM fungal infection as well as the number of OTUs increased, which also confirms that AM fungi can promote the plant to absorb nutrients. Due to the competition intensity, carbon content and fine roots are always changing with the tree growth stages; therefore, we can consider that plant growth stage is an important predictor of fungal species community.

Overall, this study showed that basic species richness and diversity were stable. The survey of the ecological distribution of AM fungi and influencing factors revealed that both biological and abiotic factors such as soil type, vegetation, geographical distance and climate could affect the AM fungal community composition. Soil type and climate determined the aeration and water content of soil, and the species diversity of AM fungi decreased significantly with the increase of flooding degree^54^. Environmental, geographical and historical factors were determinant in shaping AM fungal community^55,56^. In the course of evolution, the species richness and diversity of AM fungi changed with the evolution of host plants, and the host plants tended to choose AM fungi with high coexistence rate^57^. This study was established on a regional (county) scale that shared similar environmental conditions including soil type, climate and altitude, indicating that the AM fungal community composition varied slightly. Ecological processes structuring the fungal communities were inferred according to phylogenetic patterns and species abundance distributions^58^. Under conditions of similar climate, precipitation, altitude and niche of host plants, the AM fungal communities clustered slightly suggesting that the central process structuring communities may be driven by the growth stage of host plant.

*Cunninghamia lanceolata* generally has a rotation period of 25 years^59,60^. Considering timber yield only, the optimal rotation period can be 20 years or even 18 years^61,62^. However, studies on nitrogen deposition suggested that 30-year rotation period will be more conducive to the sustainable growth of forests^63^, some researches also recommended cutting cycles of the stands should be increased from 20–25 years to 30 years of age^64^. Our results showed that the number of shared OTUs between 32-year and 9-year, 17-year, 23-year steadily increased (from 34 to 46 and 49), while decreased in 45-year, indicating that 32-year may be a turning point in the growth of Chinese fir. The variation of *Glomus* and *Archaeospora* tended to steady state, suggesting that the 32-year rotation period may be the most favorable for microbial community.

### Effects of soil properties on AM fungal community

AM fungi have been shown to promote N nutrition and subsequently create mulches enriched in N^65^. Belowground N transfer can occur via plant-associated mycorrhizal fungi^66^, plant could control over N transformations in and near the rhizosphere by releasing root exudates^67,68^. Enhanced root exudation has been demonstrated to accelerate rhizosphere N turnover, particularly under N-limiting conditions^69^. Our research found that N was related with the AM fungal community, particularly in the 9-year. However, few studies have been done about relationships between root exudate and nitrogen enrichment of Chinese fir. The research about how root exudates influence soil N accumulation of Chinese fir through AM fungi will be a hot topic in the future.

Many previous studies have claimed that P could affect AM fungal community composition. P not only lessened AM fungal community richness and Shannon diversity in a regional scale^70^, but also affected AM fungal community diversity in a large environment^71^. Among the five stages, Chinese fir in the 9-year with a maximum AMF diversity index had minimum P content, which was 4.3 mg/kg. This phenomenon may be due to a scarcity of available P. Meanwhile, P availability might be influenced by pH, and a strongly acidic soil (pH <5) adverse to P transferring.

As a macronutrient, enzyme cofactor, and a component of chlorophyll molecule, Mg plays crucial roles in photosynthesis and stabilization of nucleotides and nucleic acids. This study demonstrated that K and Mg were highlighted as important factors influencing the AM fungal community compositions. The result was consistent with the other studies. some studies emphasized out that Mg and K were variables in the structure of the AM fungal community^72^. They also explored the relationship between the number of spores, the diversity of the AM fungal genera and some chemical soil conditions, and found Mg and K were highly correlated with the density of spores^73^. Meanwhile, AM fungal species distribution was positively related to soil properties, especially soil Mg contents^74^. More researches were concerned with the effects of AM fungal infection on Mg and K contents in plants. Compared with the control, the plant with additional AM fungi inoculation exhibited significant differences in nutrient uptake^75^, plants treated with AM fungi possessed higher K and Mg contents in the roots^76^. However, it was also shown that AM fungi inoculation had no positive influence on the contents of K and Mg^77^.

## Conclusions

There were 76 OTUs were retained from the 60 root samples of Chinese fir with Illumina sequencing. The genera covered by OTUs were *Glomus*, *Archaeospora*, *Acaulospora*, *Gigaspora* and *Diversispora*, among those *Glomus* occupied the majority(75%), followed by *Archaeospora*. The number and composition of OTUs varied along with the host plant growth. The number of OTUs showed an inverted V-shaped change with the host plant age, and the maximum occurred in 23-year. When it comes to shared OTUs, the number of shared OTUs between 32-year and 9-year, 17-year, 23-year, 45-year firstly increased then decreased, and the peak of that was in 32-year vs 23-year. There were 15 unique OTUs which only appeared in one growth stage. The proportion of each genus in 23-year was different with that of other stages. Non-metric multi-dimensional scaling (NMDS) analysis based on bray-curtis distance showed that AM fungal community in 9-year was significantly differed with that of other stages. Besides, AM fungal community in 32-year had a significant difference with that of 23-year, while no significant difference with that of 45-year, suggesting that 32-year may be a steady stage for AM fungi associated with Chinese fir. The cutting age in 32-year may be the most favorable for microbial community. Available N, available P, K and Mg were significantly related with AM fungal community based on redundancy analysis.

## Supplementary information

revised manuscript
