# Supplementary material for: Succession in arbuscular mycorrhizal fungi can be attributed to a chronosequence of Cunninghamia lanceolata
Source: Sci Rep. 2019 Dec 2;9:18057. doi: 10.1038/s41598-019-54452-z (PMC6889488; doi:10.1038/s41598-019-54452-z)
Supplement: Supplementary file 1 — revised manuscript [file 41598_2019_54452_MOESM1_ESM.doc]

**Supplementary information**

Succession in arbuscular mycorrhizal fungi can be attributed to a chronosequence of *Cunninghamia lanceolata*

**Nini Lu^1,2^, Xuelei Xu^1,2^, Ping Wang^1,2^, Peng Zhang^1,2,3^, Baoming Ji^1^, Xinjie Wang^1,2^**

^1^College of Forestry, Beijing Forestry University, Beijing 100083, China

^2^Key Laboratory for Silviculture and Conservation Joint-constructed by Province and Ministry of Education, Beijing Forestry University, Beijing 100083, China

^3^Experimental Forest Farm, Beijing Forestry University, Beijing 100095, China

*Corresponding authors: Xinjie Wang

Tel: +86-10-62336082; Fax: +86-10-62336082

E-mail: [xinjiew@bjfu.edu.cn](mailto:xinjiew@bjfu.edu.cn)


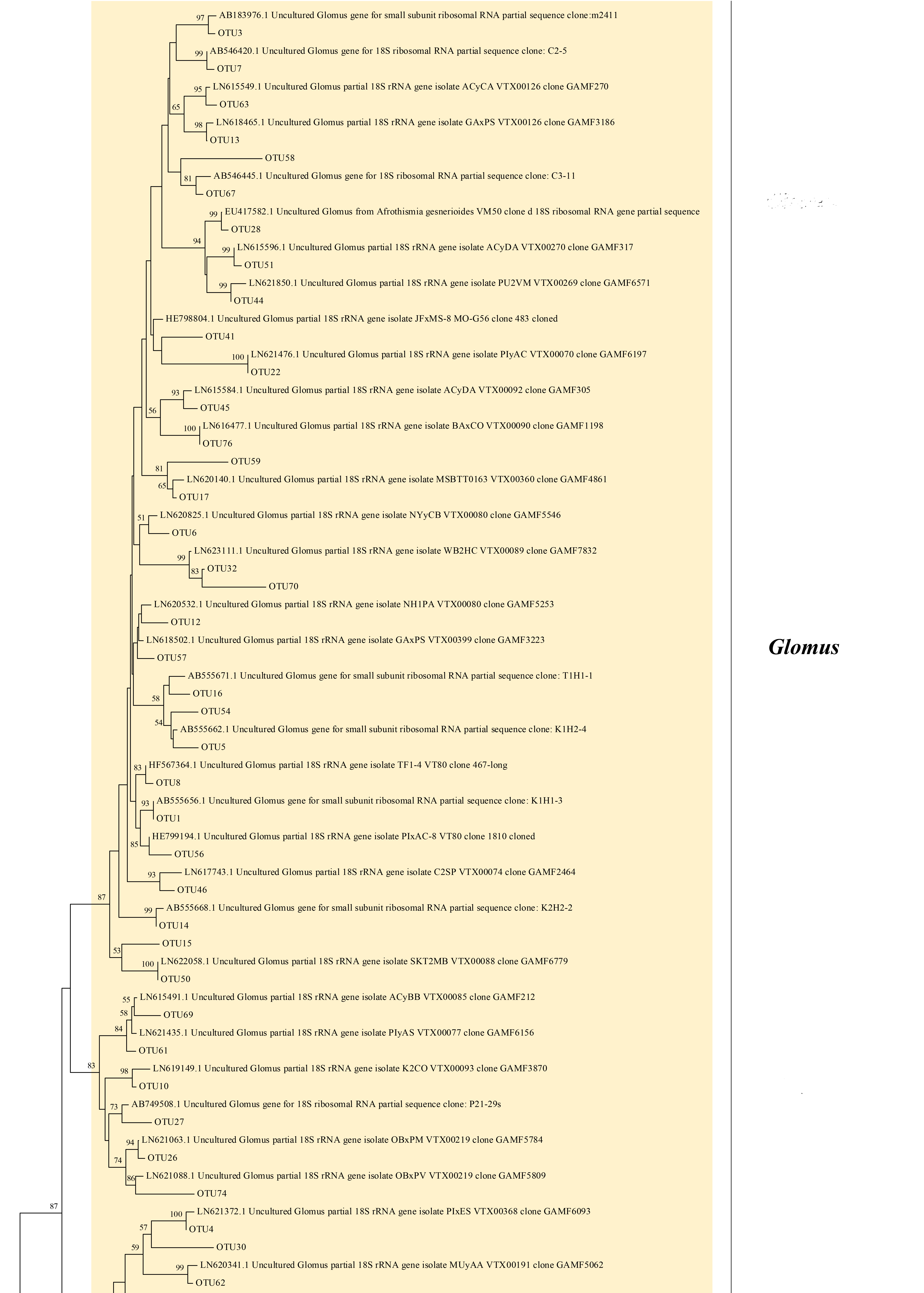

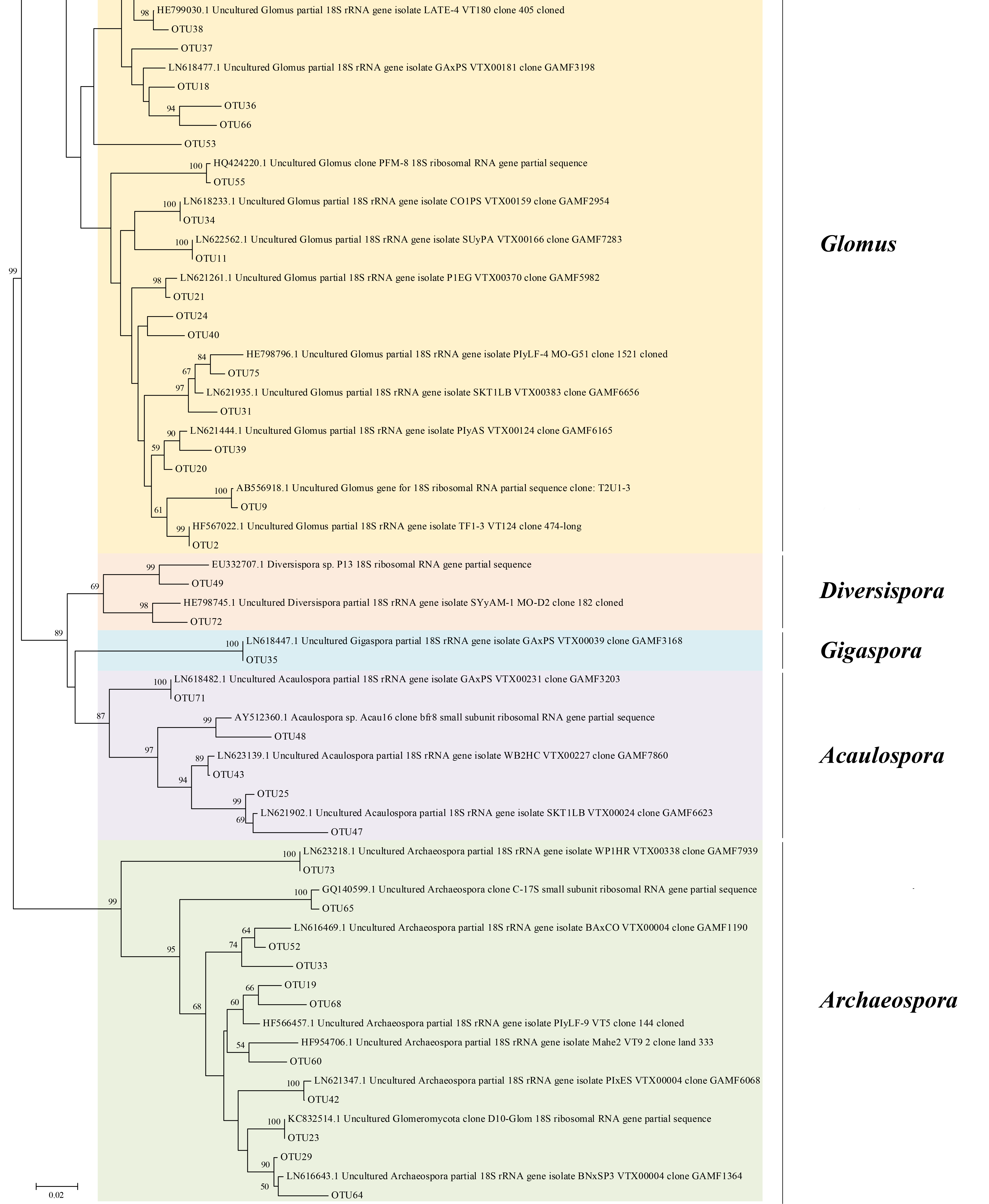


**Figure A1.** Neighbor-joining phylogenetic tree of representative sequences of each OTU detected in the study. Representative sequences are named as OTU (1-76). Different coloration of the phylogenetic tree represents different genera of AMF. Bootstrap values higher than 50% are indicated.

**Table A1.** The results of anosim testing the pairwise differences in AMF communities between the host plants’ stages.

| Stage pairs | Observed Delta | Expected Delta | *P* |
| --- | --- | --- | --- |
| Young/Adolescent | 0.56 | 0.68 | 0.001 |
| Young/Near mature | 0.59 | 0.70 | 0.001 |
| Young/Mature | 0.54 | 0.67 | 0.001 |
| Young/Over-aged mature | 0.55 | 0.68 | 0.001 |
| Adolescent/Near mature | 0.56 | 0.57 | 0.2 |
| Adolescent/Mature | 0.51 | 0.51 | 0.42 |
| Adolescent/Over-aged mature | 0.52 | 0.54 | 0.04 |
| Near mature/Mature | 0.54 | 0.56 | 0.041 |
| Near mature/Over-aged mature | 0.55 | 0.57 | 0.022 |
| Mature/Over-aged mature | 0.50 | 0.50 | 0.43 |
